# Injectable Aesthetic Treatments for Improving Facial Skin Quality in Transgender Patients With or Without Gender‐Affirming Hormone Therapy

**DOI:** 10.1111/jocd.70318

**Published:** 2025-08-23

**Authors:** Bianca Viscomi, Katherine Goldie, Martina Kerscher

**Affiliations:** ^1^ Bianca Viscomi Dermatologia Private Practice São Paulo Brazil; ^2^ Clinic 77 London UK; ^3^ Division of Cosmetic Science University of Hamburg Hamburg Germany

**Keywords:** calcium hydroxylapatite, Cohesive Polydensified Matrix hyaluronic acid, combined treatment, gender‐affirming care, hormone therapy, incobotulinumtoxinA, regenerative aesthetics, skin quality, transgender

## Abstract

**Background:**

Specialized medical care for transgender patients during the transition process can substantially improve mental and physical health for this population, due to the multitude of changes they may experience in both areas. Minimally invasive aesthetic procedures (MIPs) on the face can promote the anatomical changes and skin quality improvements necessary for gender affirmation, increasing gender congruence and, consequently, improving patients' quality of life. Given that gender‐affirming hormone therapy (GAHT) impacts skin quality, MIPs may offer valuable complementary benefits during the transition process.

**Objective:**

Here we describe a novel treatment plan to improve skin quality in transgender patients who are or are not undergoing GAHT.

**Method:**

The combination of incobotulinumtoxinA (Xeomin; Merz Pharmaceuticals GmbH, Frankfurt, Germany), calcium hydroxylapatite‐carboxymethylcellulose (Radiesse; Merz North America Inc., Franksville, WI, USA), and Cohesive Polydensified Matrix hyaluronic acid with glycerol (CMP‐HA20G, Belotero Revive; Anteis S.A., Plan‐les‐Ouates, Switzerland, a company of the Merz Aesthetics group) should be administered in a single session to improve skin quality. Photographs were taken at baseline and follow‐up to assist with 3‐dimensional reconstructions for evaluation of skin quality according to the four emergent perceptual categories (EPCs) defining skin quality.

**Outcomes:**

EPC monitoring may help to guide MIP treatments, support future evaluation of treatment efficiency, and assist in delivering personalized gender‐affirming care.

**Conclusion:**

This new treatment plan for skin quality improvement offers gender‐affirming care to the transgender community, based on clinical expertise.

## 1 Introduction

Up to 0.6% of adults in the United States identify themselves as transgender, and up to 4.5% of adults worldwide identify themselves as transgender or one of the more expansive definitions, including non‐binary and nonconforming [1, 2]. This number has increased because of growing awareness and acceptance of non‐binary genders. Yet, patients who are gender diverse still face more health disparities than those who are cisgender [2, 3, 4, 5]. Barriers to healthcare within this community include discrimination, marginalization, and limited access to providers who have both the knowledge and cultural competence necessary for transgender health [3, 6].

Medical transition assistance significantly enhances the health and life expectancy of this patient population by fostering social acceptance. This acceptance can lead to better overall well‐being and improved life expectancy. By facilitating medical transitions, such assistance helps patients gain societal acceptance, potentially shielding them from violence and contributing to a longer life [5, 6]. It is paramount that health care providers are knowledgeable and trained in medical transition assistance to care for this population effectively [3, 6].

Gender‐affirming medical care for transgender patients is multifaceted, often incorporating gender‐affirming hormone therapy (GAHT), surgeries, and minimally invasive procedures (MIPs) [3, 4, 6]. Patients who are uncertain about, or have limited access to more‐permanent procedures often pursue MIPs or reversible treatments, which are more affordable and often lower risk. MIPs include aesthetic treatments designed to improve skin quality, a reported concern in the transgender population, which can significantly impact gender identity [6]. Specifically, skin quality affects an individual's perceived health, age, and attractiveness, and can influence social perception of gender [6, 7]. Treatments that improve skin quality can positively impact self‐perception, enhance social skills, reduce anxiety and depression, and increase quality of life [8]. Skin quality is impacted by multiple factors in transgender patients. Basal and GAHT‐induced hormone levels interfere with skin quality; higher levels of masculinizing hormones are associated with increased sweat production, sebaceous gland enlargement, and increased sebum secretion, while feminizing hormones typically reduce sebum production but may lead to xerosis [3, 4, 9, 10, 11, 12, 13]. Accordingly, in a retrospective cohort study of 46 507 gender‐diverse adults, including transgender individuals, the incidence and severity of acne in patients receiving masculinizing GAHT were substantially higher than those in cisgender women and cisgender men [10]. In contrast, transfeminine patients taking feminizing GAHT typically show reduced prevalence of acne because of reduced sebum production [12].

According to expert consensus from an international panel of dermatologists and aesthetic physicians, skin quality can be described by four emergent perceptual categories (EPCs): skin tone evenness, skin surface evenness, skin firmness, and skin glow [7]. EPCs were identified to describe skin quality across all races, ethnicities, genders, and ages and were consistent across Asian Pacific, European, Middle Eastern, African, Latin American, and North American regions. EPCs have a variety of parameters that can be measured and targeted with minimally invasive aesthetic treatments (Table 1) [7]. Addressing EPCs of skin quality with MIPs allows alignment of personal features with the patient's gender identity and can greatly improve skin quality. In some cases, improving skin quality may reverse or improve undesirable effects resulting from GAHT, such as xerosis and pruritus in patients taking estrogens, scarring from acne vulgaris, or pseudofolliculitis barbae secondary to facial hirsutism in patients taking testosterone [3, 4, 6, 11, 13]. This may be especially relevant to the transgender community, as many individuals undergo GAHT (Table 2).

**TABLE 1:** Parameters of the Emergent Perceptual Categories of Skin Quality [7].

EPC	Parameters
Skin tone evenness	Pigmentation measured as amount of melanin Erythema or redness Coloration/discoloration
Skin surface evenness	Pore size Crepiness, the degree to which skin is thin or resembles crepe paper Wrinkles and lines Scars Hair Clarity as assessed by presence of black heads, white heads, pimples, or spots
Skin firmness	Elasticity Tautness/tightness Hydration
Skin glow^a^	Radiance Brightness Vibrance Luminosity Complexion

Abbreviation: EPC, emergent perceptual category.

^a^ Skin glow does not have individual parameters but can be described based on the listed terms.

**TABLE 2:** Proposed EPC‐targeted aesthetic treatments in transgender patients.

Treatment	Primary target EPC	Secondary effects across EPCs
IncoBoNT‐A (Xeomin)	Surface Evenness (pores) [6, 14]	Glow (sebaceous control) [6, 14]
CaHA‐CMC (Radiesse)	Firmness (collagen, elasticity) [6, 15]	Surface Evenness (texture) [6], Tone (pigmentation, erythema) [15]
CPM‐HA20G (Belotero Revive)	Glow (hydration, vibrance) [16]	Firmness (hydration‐dependent elasticity), Surface Evenness (roughness) [16]
Combination (all 3)	Holistic Skin Quality Enhancement [7]	Multidimensional improvements supporting gender congruence

*Note:* Primary effects reflect the most prominently targeted EPC(s) for each treatment. Secondary effects indicate additional benefits observed across other EPCs. All treatments were administered in a single session in the following order: intradermal incoBoNT‐A, followed by CaHA‐CMC, then CPM‐HA20G. EPCs include Skin Tone Evenness, Surface Evenness, Firmness, and Glow as defined by Goldie et al. 2021.

For transgender individuals in the transitioning process, skin quality is of high importance for gender perception [6]. Although MIPs can improve hormone‐induced changes to skin quality, there are currently no reported data on skin quality or treatment protocols available for transgender individuals receiving MIPs [7]. Additionally, no data are available on the impact of hormone therapy on the efficacy of MIPs to improve the skin quality of transgender individuals. Given the relationship between gender‐affirming medical care, mental health, gender dysphoria, and quality of life, treatment plans tailored to the unique needs of transgender individuals may significantly enhance their lived experience [2, 3, 8, 11, 17, 18]. Furthermore, such treatment plans may be implemented in clinical studies to evaluate the effects of endogenous and GAHT‐induced hormone levels on skin quality and treatment outcomes. Here, we present a novel injectable treatment plan designed to improve skin quality in transgender individuals, to be evaluated in future clinical application.

## 2 Methods

To improve skin quality in transgender individuals, regardless of GAHT status, this plan outlines the combined use of incobotulinumtoxinA (incoBoNT‐A; Xeomin; Merz Aesthetics), calcium hydroxylapatite‐carboxymethylcellulose (CaHA‐CMC; Radiesse; Merz Aesthetics), and Cohesive Polydensified Matrix hyaluronic acid CPM‐HA20G (Belotero Revive; Merz Aesthetics). These treatments should be administered during a single session, following their respective established protocols [6, 14, 19, 20]. The recommended plan is as follows: firstly, intradermal injections of incoBoNT‐A, followed by regenerative biostimulation with CaHA‐CMC, and finally, injectable hydration with CPM‐HA20G.

**FIGURE 1:**
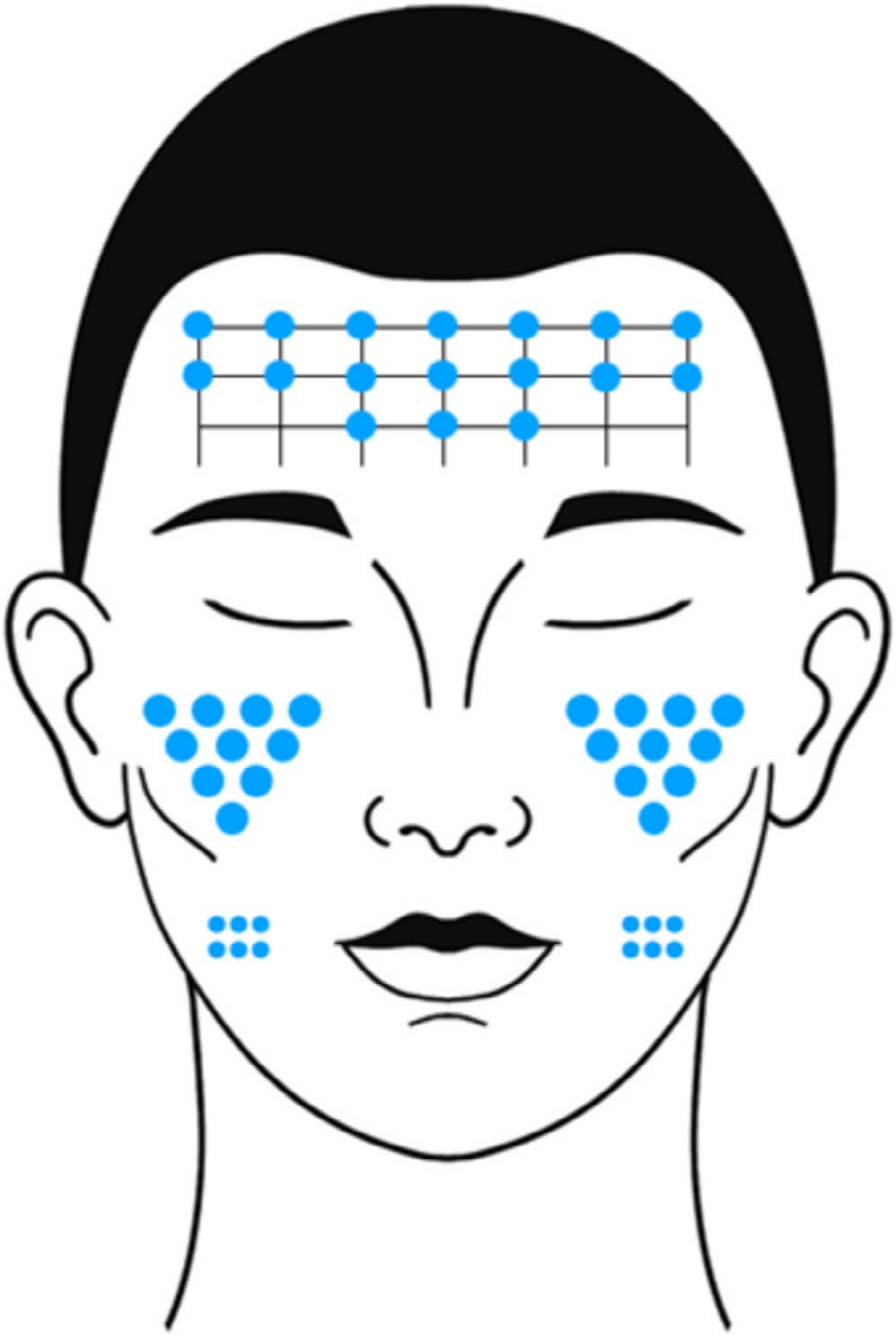
Injection procedure for incoBoNT‐A. A 100‐U vial is reconstituted with 5 mL saline for cheek and premasseteric areas, injected intradermally at 0.5 cm intervals (0.5 U per point) [14]. 100 U is reconstituted with 2 mL saline for the forehead and injected using the “One21” technique [6, 21]. Sites and doses may vary. IncoBoNT‐A = incobotulinumtoxinA (Xeomin, Merz Pharmaceuticals GmbH, Frankfurt, Germany).

For injection of incoBoNT‐A into the anterior cheek and premasseteric area (Figure 1) [6, 14, 21], each 100‐U aliquot of incoBoNT‐A is reconstituted with 5 mL of sterile saline. The anterior cheek and premasseteric area are delimited, and injections (0.5 U each) are placed intradermally 0.5 cm apart [14]. For the forehead, each 100‐U aliquot of incoBoNT‐A is reconstituted with 2 mL sterile saline, and injections are performed using the “One21” technique [6, 21]. The dose per injection point varies per patient.

**FIGURE 2:**
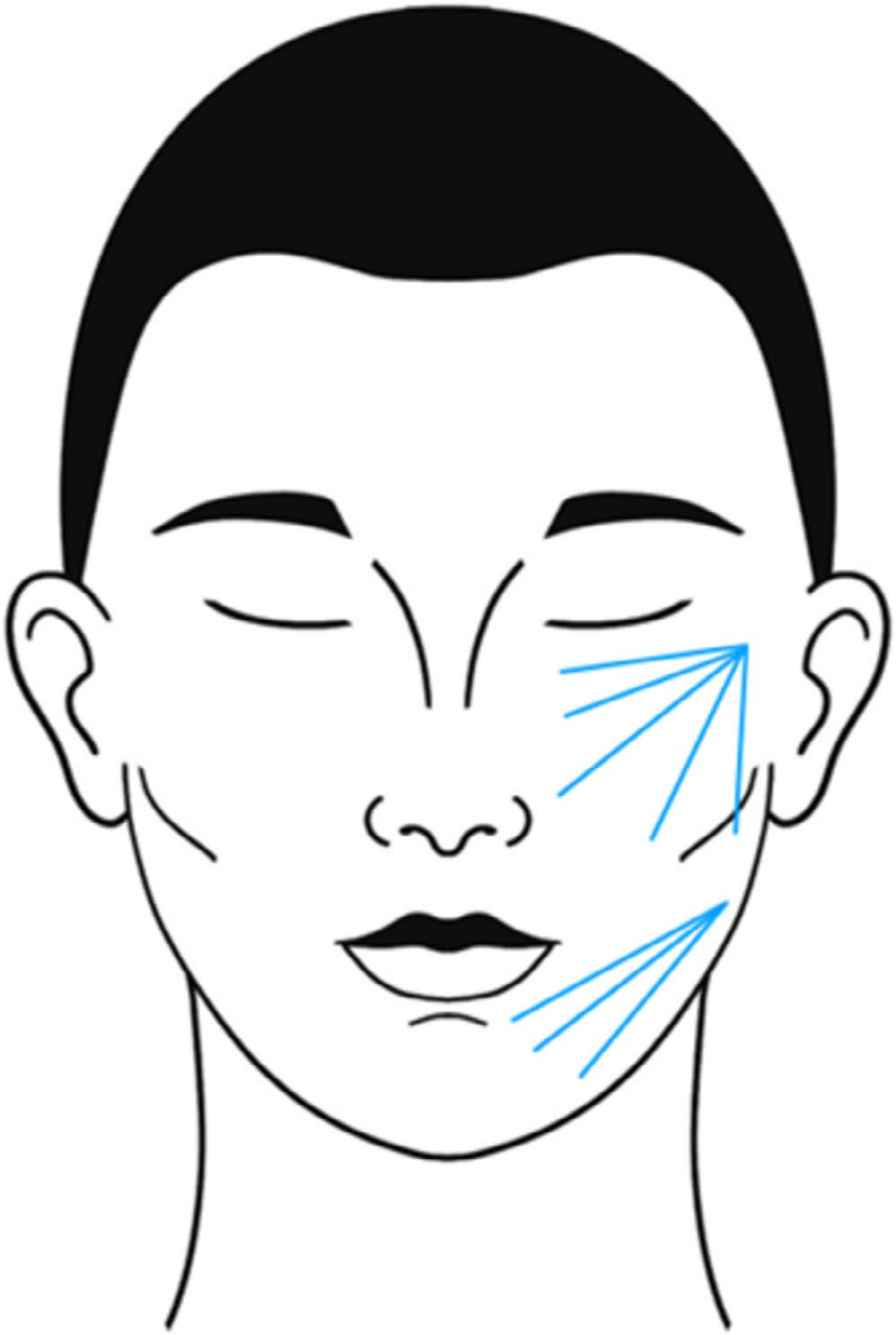
Injection procedure for CaHA‐CMC. A 1.5 mL volume is diluted 1:1 with lidocaine and saline (total 3 mL), then injected per side into the subdermal malar and premasseteric areas via retrograde microbolus technique using a 22‐ or 25‐gauge cannula [6, 19]. Product volume may vary. CaHA‐CMC = calcium hydroxylapatite‐carboxymethylcellulose (Radiesse, Merz North America Inc., Franksville, Wisconsin).

For the CaHA‐CMC injection (Figure 2) [16], 1.5 mL of CaHA‐CMC is diluted 1:1 (CaHA‐CMC: diluent) with 0.5 mL lidocaine and 1.0 mL saline (3 mL total). A total of 1.5 mL of the solution should be injected into each side of the middle and lower thirds of the face via microbolus retrograde injections along traced vectors using a 22‐ or 25‐gauge cannula [16].

**FIGURE 3:**
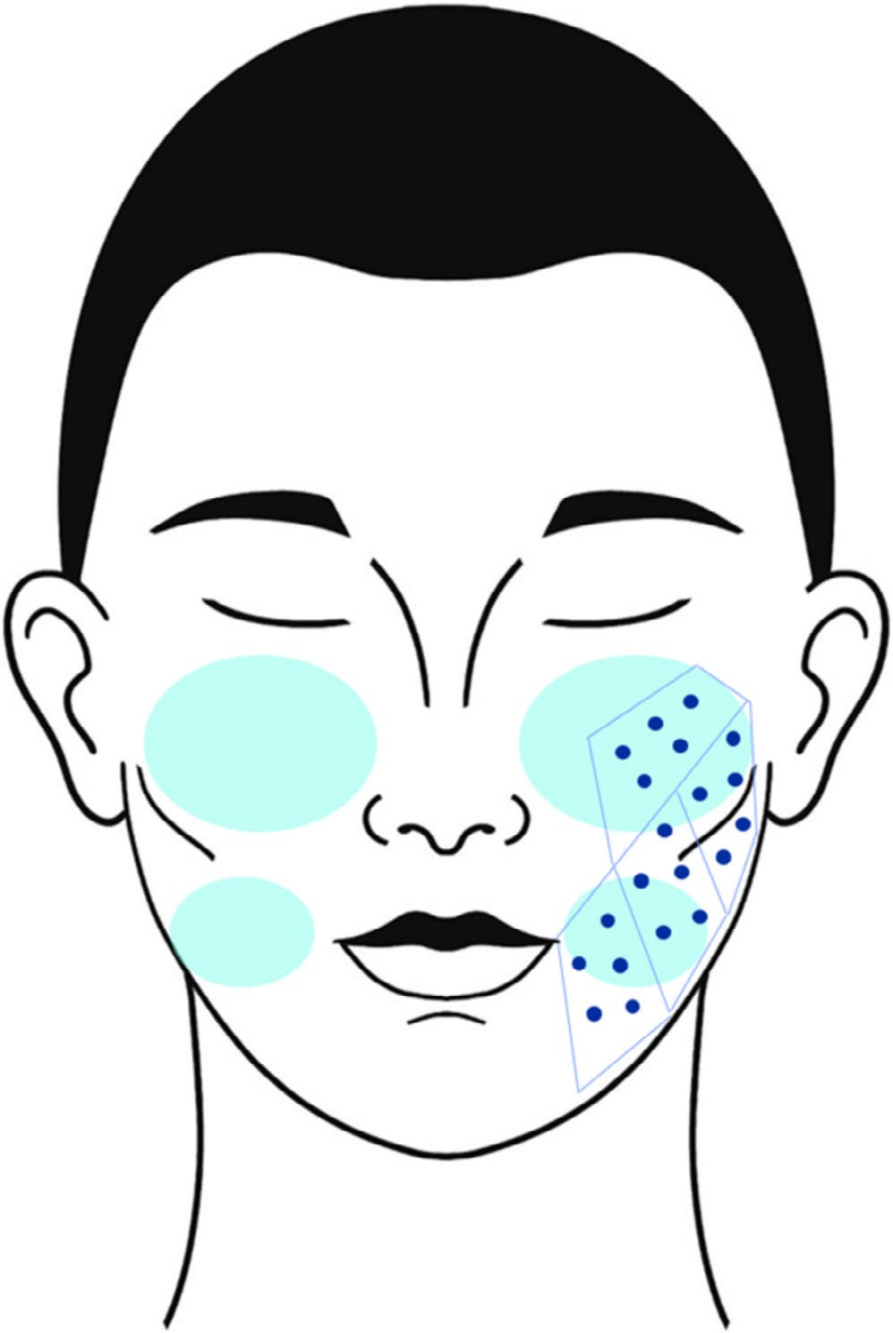
Injection procedure for CPM‐HA20G. A total of 1 mL is injected per cheek/premasseteric area using a 20‐point grid (50 μL per point) with a 32‐gauge needle into the immediate subdermis [16, 20]. Depth must be precise. CPM‐HA20G = Cohesive Polydensified Matrix hyaluronic acid (Belotero Revive, Anteis S.A., Plan‐les‐Oates, Switzerland).

For injection of CPM‐HA20G (Figure 3), a total of 1 mL is to be distributed to each cheek and premasseteric area using a needle across a 20‐injection point grid [22]. Careful attention should be paid to injection depth to avoid overly superficial administration, ensuring the product is delivered in the immediate subdermis aligned with a grid of injection points.

Patients should be evaluated and photographed at baseline and follow‐up (30‐, 90‐, and 180‐days post‐treatment) using a 2‐dimensional (2D) digital camera under standardized lighting, aperture, shutter speed, and camera distance to monitor outcomes. The resulting 2D images are then reconstructed into 3D using an imaging system (e.g., Vectra software; Canfield Scientific). Evaluations are performed based on the Scientific Assessment Scale of Skin Quality, Merz Aesthetics Scale (MAS) for upper cheek fullness, and MAS for lower cheek fullness [22, 23]. Measurements of pore count, sebum production, skin sagging, skin hydration, and skin elasticity should be assessed using 3‐dimensional imaging, sebometer, snap test, dermometer, and cutometer, respectively, at each time point and compared with baseline assessments.

## 3 Anticipated Outcomes

The 4 EPCs of skin quality provide a means to monitor skin quality in transgender patients and evaluate the efficacy of combined incoBoNT‐A, CaHA‐CMC, and CPM‐HA R treatments. The EPCs can be used in transgender patients who are or are not receiving GAHT. For instance, incoBoNT‐A can improve skin surface evenness by decreasing pore size and reducing superficial wrinkles and lines, and skin glow by reducing sebaceous gland activity [14, 24, 25]. In addition, the reduction of sebum can contribute to the improvement of acne conditions aggravated by androgenic therapy in transgender male patients [3, 4, 12]. CaHA‐CMC improves surface evenness by promoting regeneration of structural components of the skin's extracellular matrix, such as collagen, elastin, and proteoglycans [15, 26, 27, 28], thus reducing wrinkles, fine lines, and roughness [29]. CaHA‐CMC also addresses skin tone evenness by reducing pigmentation, erythema, and coloration, and increasing firmness by improving skin elasticity [7]. Further, at undiluted concentrations, CaHA‐CMC can provide immediate volumization, substantially reducing time to achieve gender‐affirming effects [15, 30]. Finally, CPM‐HA20G can safely improve skin firmness, elasticity, roughness, tone, hydration, and glow, demonstrating its ability to target all EPCs of skin quality [7, 15, 16, 30].

## 4 Discussion

Transgender people frequently face significant barriers to accessing gender‐affirming medical care, contributing to broader health disparities [17, 18, 31]. Gender‐affirming medical care, including GAHT, plays a vital role inaligning physical appearance with gender identity and improving overall well‐being [17, 32]. While GAHT can alleviate gender dysphoria and improve mental health, it may also negatively impact skin quality, presenting a therapeutic challenge [8, 17, 18, 32].

MIPs, such as incoBoNT‐A, CaHA‐CMC, and CPM‐HA20G, have emerged as accessible options that can enhance facial appearance and improve skin quality [6, 7]. They also may address specific cutaneous side effects of GAHT, such as increased sebaceous secretion in androgenic therapies and xerosis in estrogenic therapies [3, 4, 9, 12]. These interventions not only improve skin quality but may also help patients achieve greater gender congruence [7].

The four EPCs of skin quality provide a structured framework to evaluate the impacts of MIPs and tailor treatment plans to patient‐specific needs [7]. IncoBoNT‐A, CaHA‐CMC, and CPM‐HA20G each target different aspcts of skin quality and possess other favorable characteristics. For example, IncoBoNT‐A is a highly purified BoNT‐A that contains only the active 150‐kDa core neurotoxin molecule without complexing proteins and other bacterial remnants, thus resulting in a low immunogenic potential [7, 24, 33, 34, 35]. This is especially relevant for intradermal applications involving dense dendritic cell populations in the dermis that initiate the immune cascade [7, 14, 36, 37, 38, 39, 40].

CaHA‐CMC is a biostimulator that induces the regeneration of multiple extracellular matrix proteins after direct contact between CaHA microspheres and dermal fibroblasts, with effects observed at various concentration levels [15, 28, 41, 42]. CPM‐HA20G is a cross‐linked sodium hyaluronate which includes enhances hydration and supports protein synthesis, such as collagen and elastin [16, 30]. The combination of these treatmens supports broad, holistic skin quality improvement across the EPCs: tone evenness, surface evenness, firmness, and glow [7].

These injectable treatments may be preferred over surgical alternatives for some transgender patients due to the reversibility, accessibility, and effectiveness [3, 4, 6]. In addition, EPC‐based assessment allows clinicians to explore the interplay between GAHT and skin quality changes, potentially uncovering secondary effects of hormones therapy on aesthetic outcomes.

This manuscript presents a proposed treatment approach informed by clinical experience and the existing literature, but no patient data are reported. To our knowledge, this is the first proposed skin quality–focused injectable treatment plan tailored to the unique dermatologic needs of transgender patients. Clinical studies are planned to validate this protocol. Results may vary based on individual patient factors and hormone therapy status.

## 5 Conclusion

The treatment plan described herein to improve skin quality may complement other gender‐affirming care and improve quality of life [8]. Future work should evaluate outcomes of this treatment plan in transgender patients receiving care. This work will help determine whether GAHT can influence skin quality outcomes from minimally invasive aesthetic treatment. Furthermore, examination of responses to treatment in transgender patients with or without GAHT can advance understanding of the relationship between endogenous hormones, skin quality, and skin quality improvement with injectable aesthetic treatments.

## Author Contributions

All authors made equally significant contributions to the concept, design, and execution of this manuscript.

## Disclosure

B.V. is a consultant for Merz Aesthetics and is the medical director of a private practice in São Paulo, Brazil. K.G. is a consultant for Merz Aesthetics and is the medical director of a private practice in London, UK. M.K. performs clinical trials for Allergan AbbVie, Ipsen, Merz Aesthetics, and Neauvia and has served as an advisory board member or in a speaker bureau for these companies.

## Ethics Statement

No human participants or animals were involved in the manuscript. This manuscript represents original work conducted with a commitment to ethical research practices.

## Conflicts of Interest

The authors declare no conflicts of interest.

## Data Availability

Data sharing is not applicable to this article as no new data were created or analyzed in this study.
